# Understanding the cone scale in Cupressaceae: insights from seed-cone teratology in *Glyptostrobus pensilis*

**DOI:** 10.7717/peerj.4948

**Published:** 2018-06-01

**Authors:** Veit Martin Dörken, Paula J. Rudall

**Affiliations:** 1 Department of Biology, University of Konstanz, Konstanz, Germany; 2 Royal Botanic Gardens Kew, Richmond, UK

**Keywords:** Seed cone, *Glyptostrobus*, Teratology, Seed scale, Evolution, Bract

## Abstract

Both wild-type and teratological seed cones are described in the monoecious conifer *Glyptostrobus pensilis* and compared with those of other Cupressaceae sensu lato and other conifers. Some Cupressaceae apparently possess a proliferation of axillary structures in their cone scales. In our interpretation, in *Glyptostrobus* each bract of both typical and atypical seed cones bears two descending accessory shoots, interpreted here as seed scales (ovuliferous scales). The primary seed scale is fertile and forms the ovules, the second is sterile and forms characteristic tooth-like structures. The bract and the two axillary seed scales are each supplied with a single distinct vascular bundle that enters the cone axis as a separate strand; this vasculature also characterises the descending accessory short shoots in the vegetative parts of the crown. In wild-type seed cones, the fertile seed scale is reduced to its ovules, and the ovules are always axillary. In contrast, the ovules of some of the teratological seed cones examined were located at the centre of the cone scale. An additional tissue found on the upper surface of the sterile lower seed scale is here interpreted as the axis of the fertile seed scale. Thus, the central position of the ovules can be explained by recaulescent fusion of the upper fertile and lower sterile seed scales. In several teratological cone scales, the ovules were enveloped by an additional sterile tissue that is uniseriate and represents an epidermal outgrowth of the fertile seed scale. Close to the ovules, the epidermis was detached from lower tissue and surrounded the ovule completely, except at the micropyle. These teratological features are potentially significant in understanding seed-cone homologies among extant conifers.

## Introduction

Current understanding of coniferous seed-cone structure, especially the cone scale, is based partly on comparisons with extinct species known only from fossils and partly on studies of living Pinaceae extended by inference to other coniferous groups (Florin, 1951). Conifer seed cones consist of numerous overlapping structures that each represent an integrated complex combining a bract and an ovuliferous scale, here termed the bract/scale complex (Coulter & Chamberlain, 1917; Florin, 1951; Wilde, 1975; Rudall et al., 2011). The bract is a modified leaf that bears in its axil an ovulate short shoot (the seed scale or ovuliferous scale). Thus, the seed cone represents a polyaxial, branched structure (Schuhmann, 1902; Herzfeld, 1914; Pilger, 1926; Florin, 1951, 1954; Schweitzer, 1963; Mundry, 2000; Rudall et al., 2011).

In most conifers, this bract/scale complex is so tightly integrated and modified that only one integrated organ is readily discernible, termed the cone scale. Among extant conifers, the two distinct components of the cone scale are visible only in Pinaceae and Sciadopityaceae (Takaso & Tomlinson, 1991; Mundry, 2000). A factor that complicates this interpretation is that the cone scale is not strictly homologous among all conifers. For example, in Araucariaceae, the adaxial parts of the cone scale are formed by seed-scale tissues and the abaxial parts by the bract (Eckenwalder, 2009), whereas in most of the extant Cupressoideae (Cupressaceae), the bract forms the major part of the cone scale, the seed scale having been reduced to its ovules (Page, 1990; Farjon, 2007, 2010; Dörken, 2011; Groth et al., 2011; Jagel & Dörken, 2014, 2015; Dörken, Nimsch & Jagel, 2017).

In contrast with extant Cupressaceae s. str., the oldest known fossil cupressaceous seed cones retained a well-defined bract/scale complex with ovules inserted on the upper surface of the seed scale rather than in an axillary position (Spencer et al., 2015). This feature remains evident in other fossil Cupressaceae (Escapa, Cúneo & Axsmith, 2008) and some extant taxodiaceous Cupressaceae s.l., in which axillary tooth-like or tongue-like lobes are formed between the ovules and the cone scales, representing the only visible vegetative parts of the seed scale (Tomlinson & Takaso, 2002; Schulz & Stützel, 2007; Jagel & Dörken, 2014). These teeth are distinctly present in seed cones of the cupressaceous subfamilies Athrotaxoideae, Cunninghamioideae and Taxodioideae but absent from other Cupressaceae s.l. (Takaso & Tomlinson, 1989, 1990, 1991, 1992; Jagel, 2002; Farjon & Ortiz-Garcia, 2003; Tomlinson & Takaso, 2002). In Athrotaxoideae and Cunninghamioideae, the teeth remain small and are no longer visible in mature cones, so in these subfamilies the basal, adaxial part of the cone scale represents the seed scale. In contrast, in Taxodioideae the teeth are basally fused to the cone scale but the distal parts are free and become strongly elongated by intercalary growth so that they exceed the bract and ultimately enclose the seed cone. The teeth develop later than the ovules, especially in *Taxodium* and *Glyptostrobus*; their number is not strictly correlated with the number of abaxial teeth and their homologies have been disputed (Tomlinson & Takaso, 2002; Jagel & Dörken, 2014).

In this paper, we focus on both typical and atypical seed cones of *Glyptostrobus pensilis*, a rare Chinese species that represents the sole extant member of a genus with an extensive fossil history (Matsumoto, Ohsawa & Nishida, 1997; LePage, 2007). We examine spontaneous teratological seed cones of *G. pensilis* that show various different types of abnormality, including an apical vegetative proliferation of the cone axis and unusually strongly swollen teeth that bear the ovules in a central position on their upper surface.

Abnormalities that occur in nature as a result of spontaneous genetic mutations are frequently heritable and thus can enhance our understanding of homology and provide evolutionary insights, especially when viewed in a comparative and phylogenetic context (Rudall & Bateman, 2003). We use a comparative morpho-anatomical investigation to determine whether these teratologies can potentially provide new insights into interpretation of the structure of taxodiaceous cupressaceous seed cones, a topic that remains controversial even today. We focus on the axillary complexes of cone scales in general and the homologies of the teeth in particular.

Spontaneous—and potentially heritable—abnormalities are relatively common in conifer cones (Bateman, Hilton & Rudall, 2011; Rudall et al., 2011). This apparent developmental plasticity potentially enables reversals to ancestral morphological character states in extant conifers. Carlsbecker et al. (2013) described a transition series from a branched short shoot to a reduced ovuliferous scale in the well-characterised *acrocona* mutant of *Picea abies* (Pinaceae). *Glyptostrobus* belongs to subfamily Taxodioideae of the conifer family Cupressaceae sensu lato, in which a range of spontaneous mutations have been already demonstrated (Dörken, 2011; Dörken & Nimsch, 2017). Cupressaceae includes 30 extant genera placed in seven subfamilies (Gadek et al., 2000; Yang, Ran & Wang, 2012), of which five were formerly segregated as a paraphyletic family Taxodiaceae (Athrotaxoideae, Cunninghamioideae, Sequoioideae, Taiwanioideae, Taxodioideae; here termed the taxodiaceous Cupressaceae).

## Materials and Methods

The rare deciduous conifer *G. pensilis* (Staunton ex D. Don) K. Koch (Taxodioideae, Cupressaceae s.l.) is a monoecious tree species in which both seed and pollen cones are produced terminally on short lateral branchlets. A total of two cone types were investigated for this paper: (1) wild-type seed cones at the stage of pollination and at maturity (60 cones in total), (2) teratological seed cones showing a range of proliferations (five teratological cones in total). All cones were collected by one of the authors (VD) from trees grown as pot plants. Wild-type seed cones were collected from a tree cultivated in the Botanic Garden of the Ruhr-University, Bochum (Germany). All proliferated seed cones were collected from a tree cultivated in the Botanic Garden of the Eberhard Karls University Tübingen (Germany).

Freshly collected material was photographed, then fixed in FAA (100 ml FAA = 90 ml 70% ethanol + 5 ml acetic acid 96% + 5 ml formaldehyde solution 37%) before being stored in 70% ethanol. Cone anatomy was studied from serial sections using the classical paraffin technique and subsequent astra blue/safranin staining (Gerlach, 1984). Macrophotography was accomplished using a digital camera (Canon PowerShot IS2) and microphotography with a digital microscope (Keyence VHX 500F) equipped with a high-precision VH mounting stand with X-Y stage and bright-field illumination (Keyence VH-S5). Scanning electron Microscope investigations were performed in the Electron Microscopy Center, Department of Biology, University of Konstanz (Germany) with an Auriga Zeiss TM.

## Results

### Wild-type seed cones (Figs. 1 and 2)

**Figure 1:**
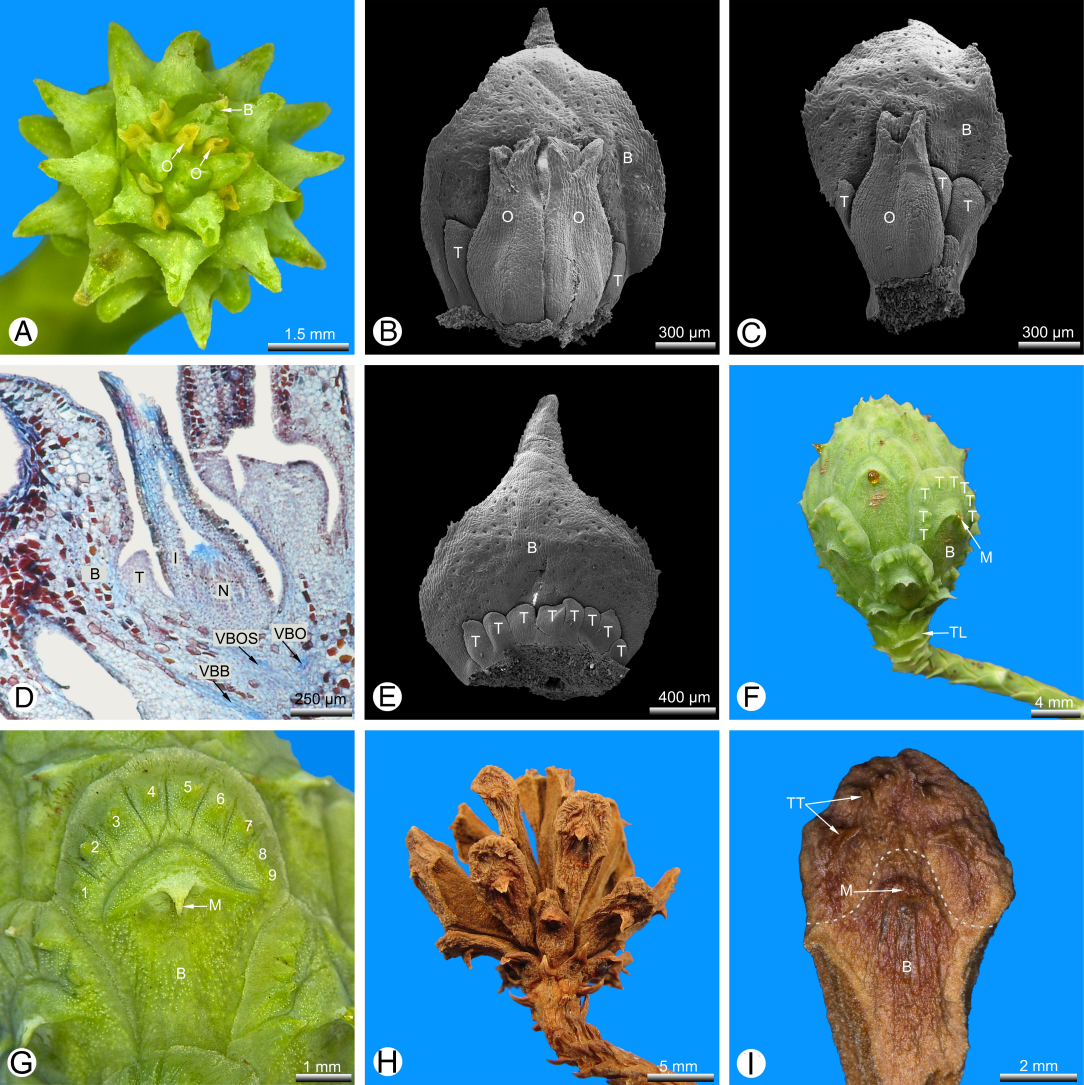
*Glyptostrobus pensilis*, wild-type seed cones at pollination (A–E), seed ripening (F and G) and seed dehiscence (H and I). (A and F–I) taken using macrophotography; (B, C and E) SEM of dissected cone scales; (D) light micrograph. (A) Entire cone from above; only the bract and micropylar tubes are visible externally. (B) Ovulate cone scale with two ovules; several abaxial teeth are present and significantly shorter than the ovules. (C) Cone scale with a single ovule and several abaxial teeth. (D) Longitudinal section of a fertile cone scale; the bract, ovule and abaxial teeth are each supplied by an individual vascular bundle. (E) Sterile basal cone scale showing only axillary teeth (ovules not developed). (F) Fertilised seed cone in lateral view. (G) Detail of a cone scale; a series of nine axillary teeth (numbered 1–9) form an arc above the bract. (H) Seed cone after release of seeds. (I) Detail of a single cone scale removed from (H); a clear boundary layer is marked as white dotted line between the bract and the distal teeth. Key: B, bract; I, integument; M, mucro; N, nucellus; O, ovule; T, tooth; TL, transitional leaf; VBB, vascular bundle of bract; VBO, vascular bundle of ovule; VBOS, vascular bundle of ovuliferous scale (= teeth). Photo credit: V.M. Dörken.

**Figure 2:**
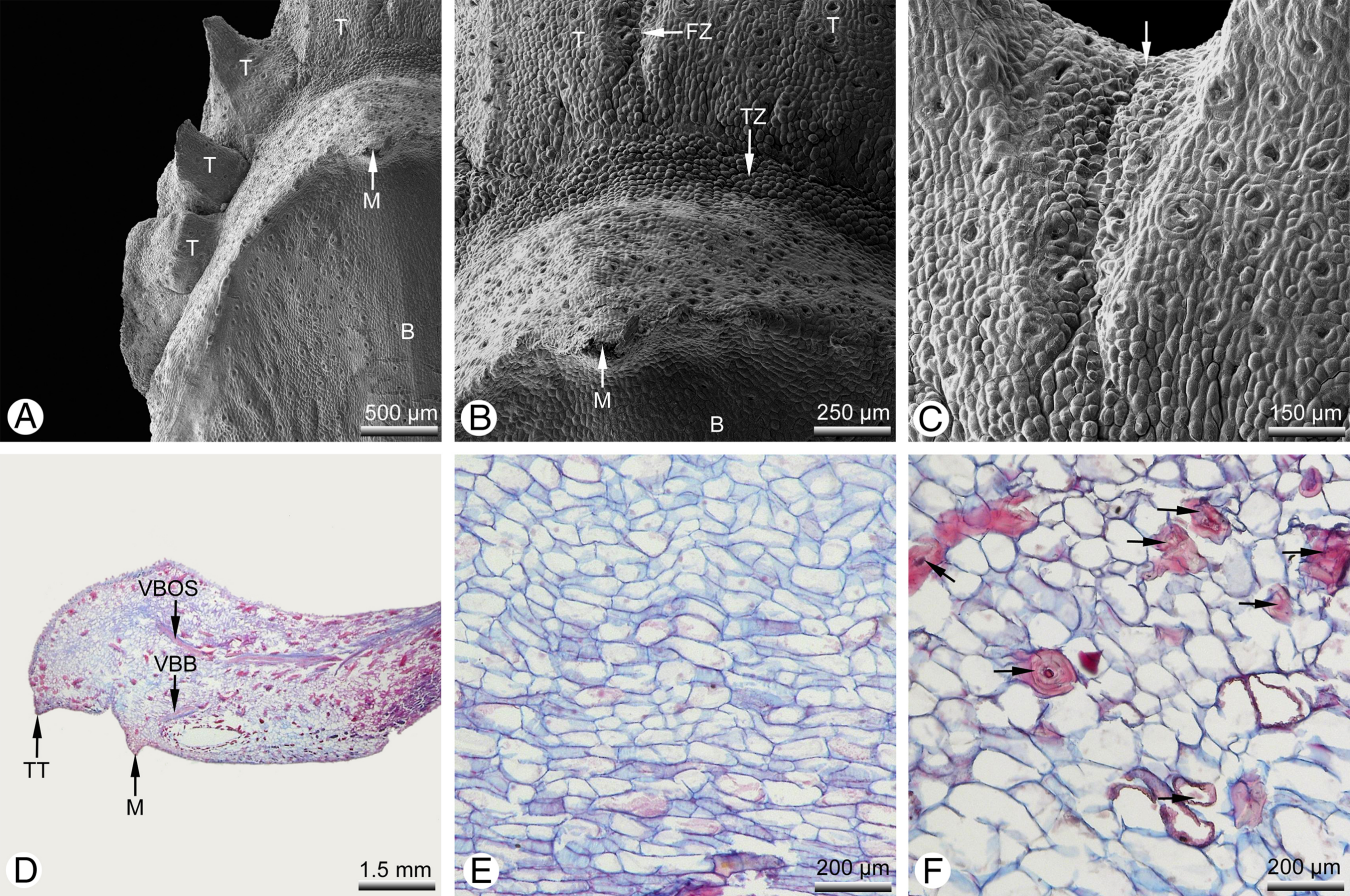
*Glyptostrobus pensilis*, morpho-anatomical details of a mature cone scale at pollination stage. (A–C) SEM; (D–F) light micrographs. (A) Distal region showing free tips of axillary teeth. (B) Distal transitional zone between the bract and the teeth. (C) Distal fusion zone (arrow) between two teeth. (D) Longitudinal section of a sterile cone scale; each scale is supplied by a distinct vascular bundle. (E) Tissue of ovuliferous scale (seed scale); the cells are parenchymatous and relatively unlignified. (F) Tissue of bract; the parenchymatous tissue is interspersed with several thick-walled sclereids (arrows). Key: B, bract; FZ, fusion zone; M, mucro; T, tooth; TT, tip of a tooth; TZ, transfusion zone; VBB, vascular bundle of bract; VBOS, vascular bundle of ovuliferous scale (= teeth). Photo credit: V.M. Dörken.

Seed cones are developed terminally on short lateral shoots and subtended by closely spaced scale-like leaves. At pollination stage, the cone is typically shaped like a spiked ball or mace (Fig. 1A) with a more-or-less plagiotropic orientation, though soon after pollination it changes to an upright position. The cone consists of several helically arranged cone scales that overlap each other (Figs. 1A, 1F and 1G). Only the cone scales in the middle of the seed cone are fertile; the basal and distal ones are sterile (Fig. 1A). Several sterile transitional leaves are present below the basal cone scales (Fig. 1F). There are mostly two ovules per cone scale (Figs. 1A and 1B), or rarely one (Fig. 1C), three were not found. At the stage of pollination, several small teeth are present below the ovules in the axial of the bract (Figs. 1B–1D). The number of abaxial teeth does not correlate with the number of ovules. In the material investigated, the number of adaxial teeth per bract varied from three to 11. Teeth are also present on the sterile distal and basal cone scales (Fig. 1F), which were described as semifertile by Takaso & Tomlinson (1990). After pollination, the teeth become strongly elongated by intercalary growth so that they finally exceed the bract in length (Figs. 1F–1I) and take part in closing the cone (Figs. 1F and 1G). At maturity, a series of outer teeth forms an arc above the bract (Figs. 1F–1H). The cone scale, axillary teeth and ovules are each supplied by an individual vascular bundle that enters the stem bundle of the cone axis as a separate strand (Fig. 1D). Stomata are irregularly but densely scattered on both sides of the bract and axillary teeth (Figs. 1B, 1C, 1E and 2A–2C).

Mature seed cones are pyriform to obovate in shape (Fig. 1F). The apex of the cone axis is used up in forming the distal cone scales. The teeth are adnate to the bract for half their length (Figs. 1F–1I) and also basally connate (Figs. 2A–2C) so that only their distal tips remain free (Fig. 2A). There is no distinct boundary between the bract and the axillary teeth (Fig. 2D), but the tissue of the bract contains many isolated sclereids (Fig. 2F), which are less well-developed in the teeth (Fig. 2E). After pollination, the bract swells so that its tip becomes shifted to the back of the bract; the tip is visible at maturity as a small backward-pointing mucro (Figs. 1F, 1G, 1I, 2A, 2B and 2D). The seed cones mature in the year of pollination and dry out to release the winged seeds. Shrinking of the cone axis and cone scales causes the cones to open and the cone scales to spread apart (Fig. 1H). At maturity, the bracts and teeth remain fused to each other (Fig. 1I).

### Teratological seed cones (Figs. 3–5)

**Figure 3:**
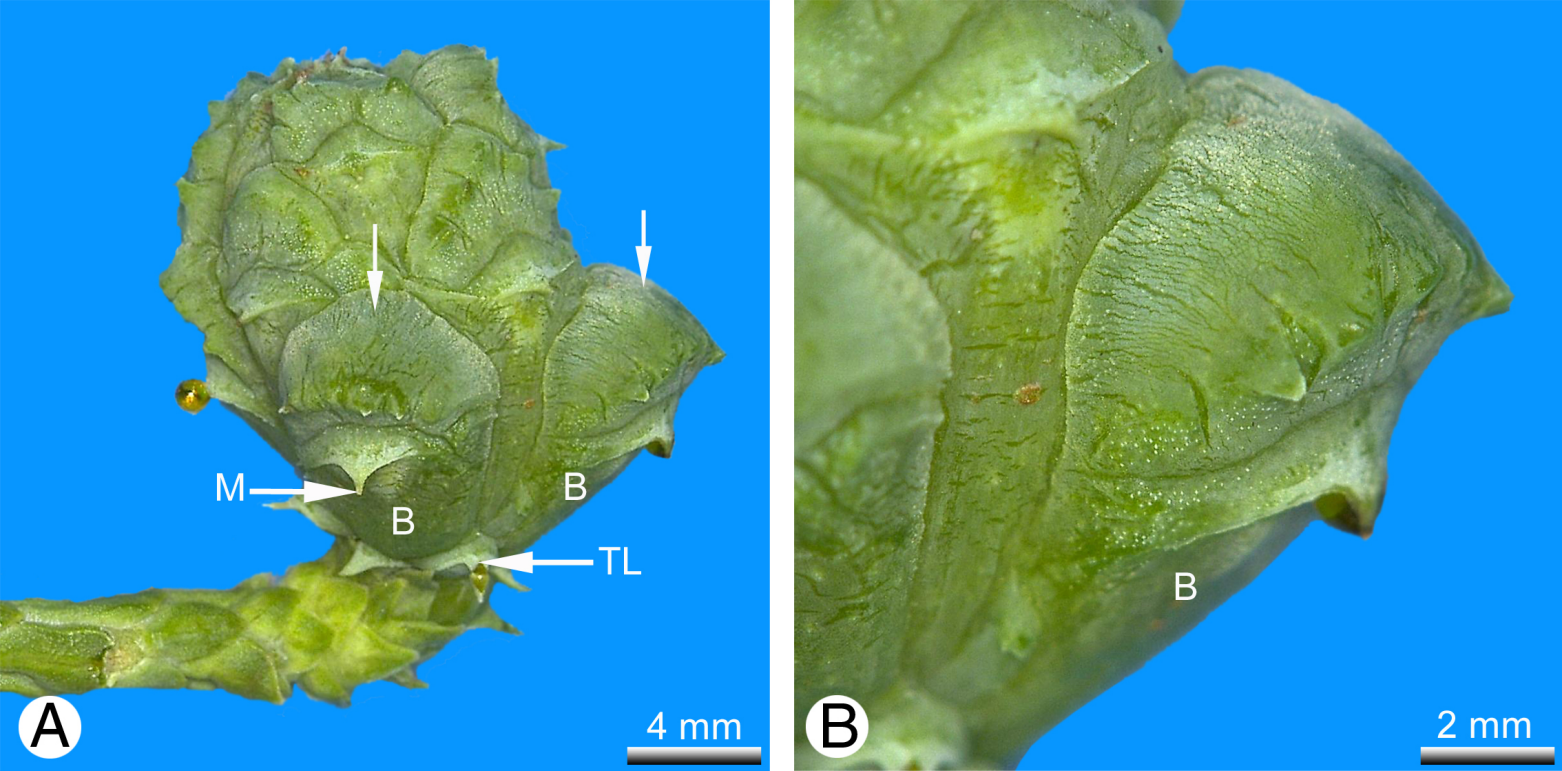
*Glyptostrobus pensilis*, teratological seed cone in which some of the cone scales show an unusually strongly swollen ventral bulge (arrowed), so that they spread away from the others. (A) lateral view; (B) detail of (A); Key: B, bract; M, mucro; TL, transitional leaf. Photo credit: V.M. Dörken.

**Figure 4:**
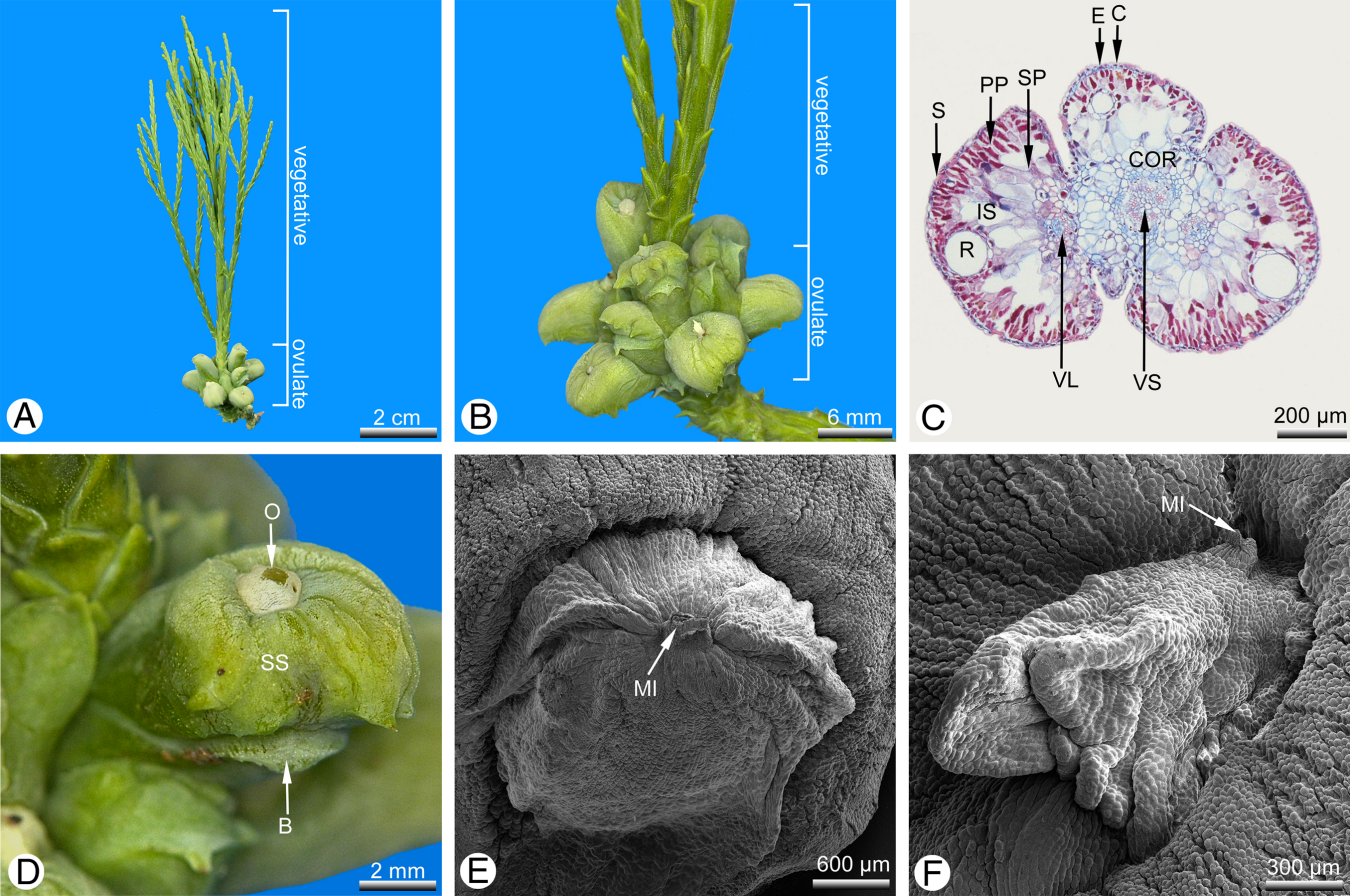
*Glyptostrobus pensilis*, teratological seed cone (cone A) in which the cone axis is strongly proliferated and the axillary teeth are unusually swollen. (A) Lateral view. (B) Detail of (A). (C) Transverse section of a branchlet in the terminal proliferation. (D) Detail of a bract/scale complex with ovule dislocated into the centre of the upper surface. (E and F) SEM details of two different dislocated, central ovules; in both cases, the ovule is enveloped by an additional tissue so that only the micropyle remains free; the enveloping tissue is wrinkled, particularly in (F). Key: B, bract; C, cuticle; COR, shoot cortex; IS, intercellular space; E, epidermis; MI, micropyle; O, ovule; S, stomata; SP, spongy parenchyma; SS, sterile tooth-bearing ovuliferous scale; PP, palisade parenchyma; R, resin duct; OS, ovuliferous scale (seed scale); VL, vascular bundle diverging to leaf; VS, vascular bundle of shoot). Photo credit: V. M. Dörken.

The teratological seed cones examined here showed two distinctly different types of anomaly: strongly swollen ovuliferous scales (Fig. 3), and apical vegetative proliferation of the cone axis (Figs. 4A and 4B).

**Figure 5:**
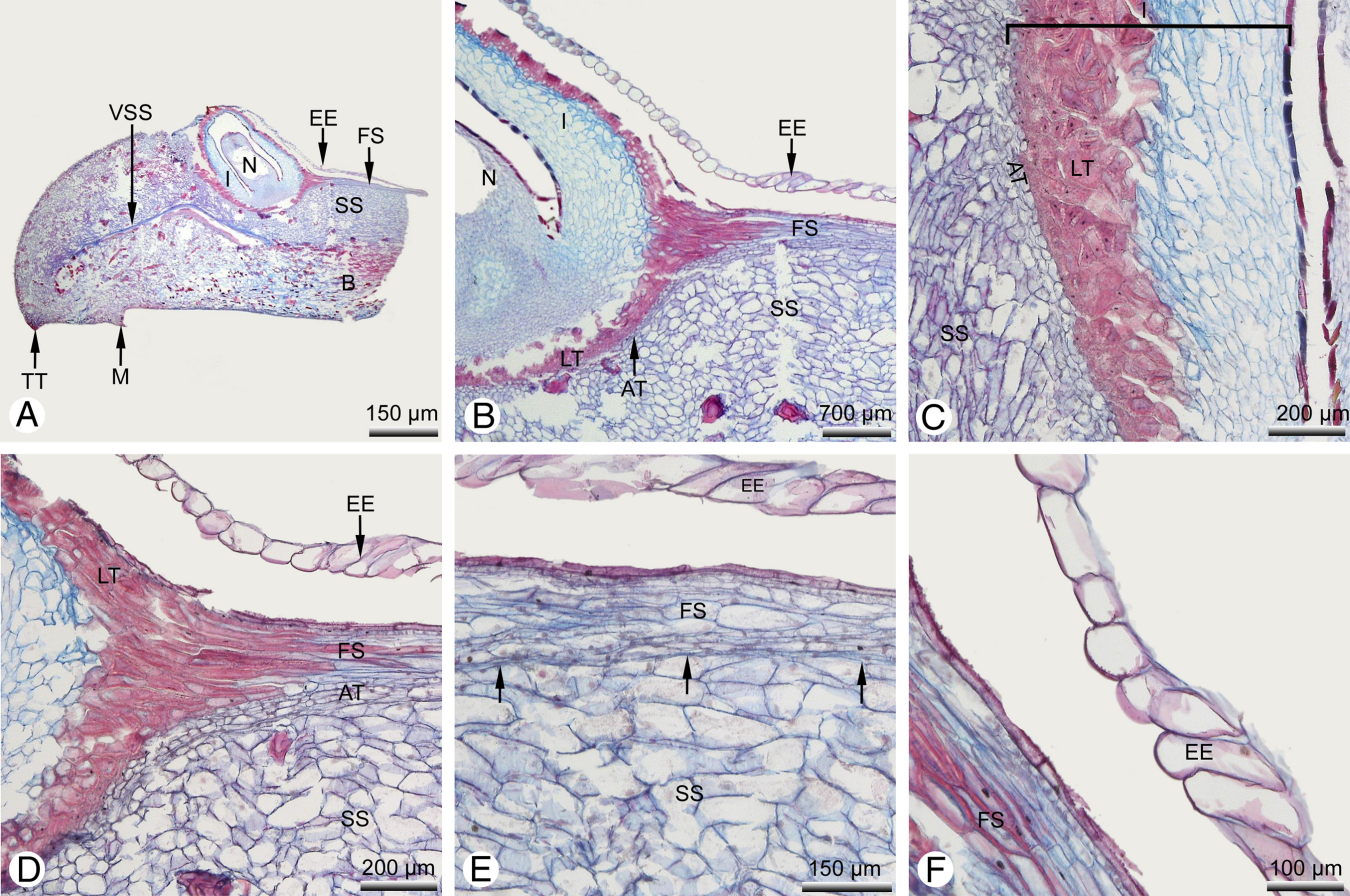
*Glyptostrobus pensilis*, anatomical details of a single cone scale taken from teratological seed cone A (illustrated in Fig. 4). (A) Longitudinal section showing strongly swollen tooth with a centrally located ovule; the nucellus is surrounded by the integument, but also by an additional epidermal tissue of the fertile ovuliferous scale. (B–D) Details of the ovule; the outer parts of the integument are already strongly sclerenchymatous, forming a typical lignified testa; an abscission tissue is present below the ovule. (E) Detail of the upper surface of the ovuliferous scale showing a clear boundary layer (arrowed) separating the tissues of the upper fertile ovuliferous scale from the lower sterile one. (F) Detail of the tissue enveloping the ovule, consisting of a single layer of non-lignified cells. Key: AT, abscission tissue; B, bract; EE, epidermal envelope; I, integument; M, mucro; N, nucellus; O, ovule; FS, fertile ovule-bearing ovuliferous scale; LT, lignified outer region of integument that forms the woody testa in the mature dry cone; SS, sterile tooth-bearing ovuliferous scale; TT, tip of a tooth; VSS, vascular bundle ovuliferous scale). Photo credit: V.M. Dörken.

In the majority, the cone scales had anomalous strongly swollen teeth that formed a large ventral bulge, causing those cone scales to spread apart from the others. As in wild-type cones, the ovules remained in their original position, deeply hidden within the cones and not dislocated by the strongly swollen abaxial tissue of the anomalous teeth. Thus, as in wild-type seed cones, in these cones the ovules are not visible externally (Fig. 3).

One remarkable teratological seed cone (designated cone A: Figs 4 and 5) possessed two different types of anomaly: strongly swollen teeth, as described above, and an apical vegetative proliferation of the cone axis (Figs. 4A and 4B). In this cone, the apex was not used up by formation of the distal cone scales (as in the wild type), but instead maintained a longer period of vegetative growth, forming a well-developed vegetative shoot axis that elongated the cone axis and even produced lateral fifth-order branchlets (Fig. 4A). The leaves inserted on the apical proliferation (Fig. 4C) corresponded in all morpho-anatomical features with the scaly trophophylls (leaves) on typical vegetative parts in *G. pensilis*; no transitional leaf types were observed in this region (Fig. 4B). In contrast, several basal leaves on seed cone A were transitional between the typical scaly trophophylls and the basal cone scales (Fig. 4B).

In cone A, each distal cone scale was fertile and bore a single ovule (Figs. 4A, 4B and 4D). The most proximal (basal) cone scales were sterile and displayed only a distinct row of axillary teeth that exceeded the bract in length (Fig. 4B). In the fertile cone scales, the axillary teeth below the ovules were unusually strongly swollen, forming a distinct ventral bulge, so that the tissue of this bulge ultimately formed the bulk of the cone scale (Figs. 4D and 5A). The ovules were not axillary (as in the wild-type) but displaced into a central position on the upper surface of the swollen teeth (Figs. 4B, 4D–4F and 5A). A further tissue belonging to a second upper fertile ovule-bearing ovuliferous scale was present from the cone axis to shortly behind the ovule (Fig. 5). A distinct boundary layer was evident between the upper fertile ovule-bearing ovuliferous scale, which consisted of flattened elongated cells, and the tissue of the lower sterile ovuliferous scale with strongly swollen teeth, which consisted of enlarged parenchymatous cells (Figs. 5B, 5D and 5E). As in the wild type, the bract, teeth and ovule were each supplied with a discrete vascular bundle, each bundle separately diverging from the concentric stem bundle of the cone axis.

In all the cone scales in cone A, the ovule was entirely covered (except at the micropyle) by an additional envelope consisting of a single layer of non-lignified cells (Figs. 4D–4F and 5A), which was not evident in the wild type. This additional layer either fitted tightly over the ovule on the integument surface (Figs. 4A, 5A, 5B and 5D–5F) or in some cone scales it was so strongly developed that it became strongly wrinkled at basal regions (Fig. 4F). In either case, it was not fused to the ovule.

## Discussion

### Comparison of *Glyptostrobus* with other taxodiaceous Cupressaceae indicates the presence of accessory shoot shoots

Our observations of wild-type seed cones of *G. pensilis* confirm that the ovules are axillary, though they later fuse with the abaxial teeth and the bract. This feature is extremely diverse in Cupressaceae. Ovules are also axillary in the other two genera of the subfamily Taxodioideae (*Cryptomeria* and *Taxodium*), but not in the monogeneric subfamilies Athrotaxoideae (*Athrotaxis*) and Cunninghamioideae (*Cunninghamia*), in which the ovules develop close to the cone axis on tongue-like structures that can be understood as representing the tips of the ovuliferous scale. Assuming that the bract is a modified leaf that bears the ovuliferous scale in its axil, as widely interpreted, the ovuliferous scale of *Athrotaxis* and *Cunninghamia* has shifted from its original axillary position by recaulescence; specifically, by adnation (fusion) of the ovuliferous scale to the subtending bract, resulting in its displacement onto the bract (Jagel, 2002; Jagel & Dörken, 2014). The other two taxodiaceous subfamilies, Sequoioideae (*Metasequoia*, *Sequoia* and *Sequoiadendron*) and Taiwanioideae (*Taiwania*), differ strongly in this respect from other taxodiaceous Cupressaceae in lacking visible parts of the ovuliferous scale and in ovule insertion close to the cone axis, always in basal regions of the upper surface of the cone scale, but never axillary. Thus, for Sequoioideae and Taiwanioideae, the ovuliferous scale is reduced to its ovules; any further visible parts that could be interpreted as vegetative outgrowths of the ovuliferous scale are invisible (Jagel, 2002; Schulz & Stützel, 2007; Jagel & Dörken, 2014).

Similarly, there are significant ontogenetic differences between the formation of the axillary teeth in Taxodioideae and the tongue-like structures of Athrotaxoideae and Cunninghamioideae. In all three genera of Taxodioideae (including *Glyptostrobus*), the teeth start to develop relatively late, significantly after ovule formation, so that they are shorter than the ovules at pollination (Takaso & Tomlinson, 1989, 1990; Jagel, 2002; Jagel & Dörken, 2014; this paper, Fig. 1). During maturation they become fused to each other and further growth occurs basal to the teeth in an intercalary fashion. Only their distal tips remain free and distinctly exceed the bract in length, so that at maturity an arc is visible above each bract, formed by a series of teeth. Thus, the teeth participate in closure of the seed cone, supporting their potential role as protective structures (Tomlinson & Takaso, 2002). In contrast, the tongue-like structures of Athrotaxioideae and Cunninghamioideae degenerate soon after pollination and are no longer visible in mature seed cones, so they do not take part in closure of the seed cone. Instead, seed cones in Cunninghamioideae become enclosed by enlarged overlapping bracts, whereas seed cones of Athrotaxioideae are enclosed by a distinct distal ventral bulge that develops on the upper surface of the bract (Jagel, 2002; Schulz & Stützel, 2007; Jagel & Dörken, 2014).

Seed-cone structure is fairly similar in all three genera of Taxodioideae, especially between *Glyptostrobus* and *Taxodium*, reflecting their close relationship (Gadek et al., 2000; Yang, Ran & Wang, 2012), but they differ in three respects: formation of the cone axis, correlation between tooth and ovule number, and tooth ontogeny. In *Taxodium* and *Cryptomeria*, the cone axis is condensed so that mature seed cones are more or less ovoid, whereas in *Glyptostrobus* the mature seed cones are relatively elongated and obovoid (Jagel & Dörken, 2014; this paper). One of the most striking differences between the three genera is the correlation between the number of ovules and the number of abaxial teeth. In *Cryptomeria*, the original one-to-one ratio is disrupted by ovule abortion (Takaso & Tomlinson, 1989), but in *Taxodium* and *Glyptostrobus* the number of abaxial teeth always significantly exceeds the number of ovules (Takaso & Tomlinson, 1990; Jagel & Dörken, 2014). The three genera also differ in the ontogeny of the teeth. In *Cryptomeria*, both the ovules and the abaxial teeth develop from the same axillary meristem-complex; at the earliest ontogenetic stages this is visible as a swelling in the axil of the bract and the ovules appear to develop first (Tomlinson & Takaso, 2002). However, it remains unclear whether the ovules and abaxial teeth represent a common organ or independent organs derived from the same primordium. In *Glyptostrobus* and *Taxodium* the teeth develop even later than in *Cryptomeria* and are not initiated until the ovule integument has already enveloped the nucellus and formed a micropyle (Takaso & Tomlinson, 1990; Jagel, 2002). Thus, these differences in development are based on a slight change in developmental regulation. To resolve this question, further detailed ontogenetic and developmental studies are needed.

Early ovule development does not contradict interpretation of the abaxial teeth as a seed scale. In one interpretation (preferred by us), each bract bears two descending accessory seed scales, one fertile and one sterile; the primary ovule-bearing seed scale is reduced to its ovules, while the second lower tooth-bearing seed scale is sterile (Jagel & Dörken, 2014). Descending accessory shoots are not unusual for taxodiaceous Cupressaceae. They can also occur in the vegetative parts of some taxa (e.g. *Metasequoia* and *Sequoia*: Sequoioideae), where they replace the abscised short shoots (Dörken & Stützel, 2009; Dörken, 2012). Although they are less clearly demarcated in seed cones, in modern Cupressaceae (e.g. *Cupressus* and *Callitris*), each axillary row of ovules can be understood as an axillary ovuliferous shoot evolutionarily reduced to its ovules. In taxodiaceous Cupressaceae, the presence of descending accessory shoots is strongly supported by the increased number of ovules compared with other conifers. In particular, Sequoioideae show very high ovule number, arranged in one (*Metasequoia*), two (*Sequoia*) or three rows (*Sequoiadendron*) inserted in the axil of the bract (Jagel & Dörken, 2014). In these species, development of the axillary rows is always centrifugal, compared with consistently centripetal development of ovules within each row. Thus, the ovulate row that is furthest from the bract develops first, followed by subsequent lower rows. This developmental sequence indicates that each of the rows is an axillary ovulate shoot reduced to its ovules, each representing a descending accessory ovulate shoot (i.e. a fertile ovuliferous scale). The descending accessory seed scales of Sequoioideae differ from those of subfamily Taxodioideae only in that they are all fertile and possess a relatively large number of ovules per ovuliferous scale.

The vasculature of the wild-type *Glyptostrobus* seed cones investigated here provides further evidence for the presence of descending accessory shoots. Three distinct vascular bundle strands are present in a typical fertile bract/scale complex (Aase, 1915; this paper, Fig. 1D). The bract, the sterile region of the seed scale (i.e. the teeth) and the fertile region of the seed scale (which is reduced to its ovules) are each supplied with their own vascular bundle. The vascular bundles are not fused to each other and enter the concentric bundle of the cone axis as separate strands. This vascular branching pattern is closely similar to the condition of descending accessory shoots in the vegetative regions, as illustrated for *Metasequoia* and *Sequoia* by Dörken (2012; Fig. 20), showing a bract carrying axillary two vegetative descending accessory short shoots. Here also, three vascular bundle strands are visible; a collateral bundle supplies the bract and two concentric bundles each supply one of the axillary descending shoots.

### Teratological seed cones support an accessory shoot hypothesis

Superficially, the non-axillary condition of the ovules in the teratological seed cones of *Glyptostrobus* described here might appear to contradict the hypothesis of descending accessory shoots. However, longitudinal sections (Fig. 5) demonstrate a remarkably distinct boundary layer between the ovulate tissue and the ovuliferous scale tissue (Figs. 5B and 5E), indicating two distinct axillary tissues. The tissue on the upper surface of the tooth bears the ovule; it extends from the cone axis and terminates at the ovule. Thus, it could be interpreted as representing an unusually elongated primary fertile seed scale that is fused for its entire length to the lower second (sterile) seed scale, which forms the teeth (Fig. 5A).

**Figure 6:**
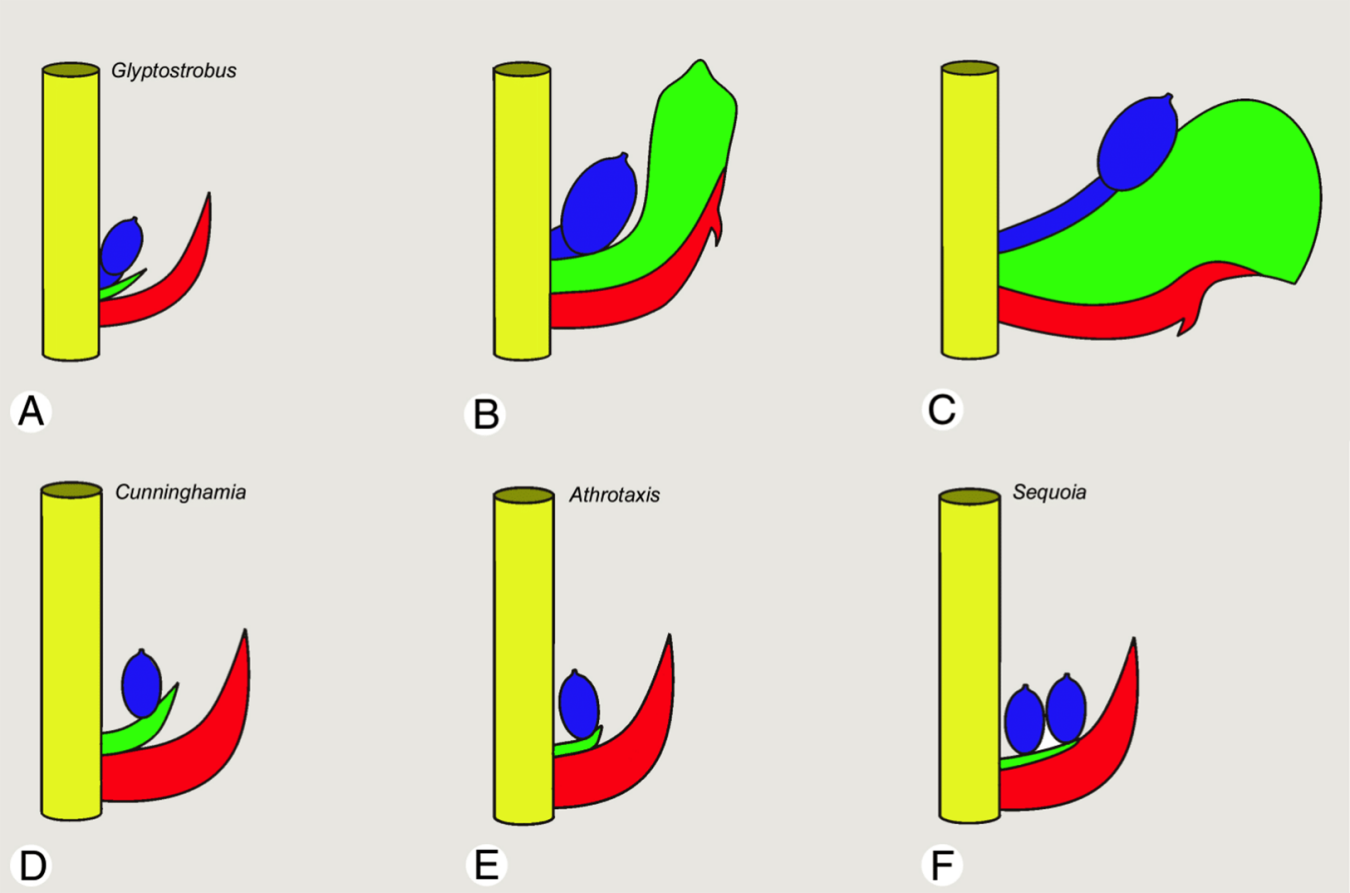
Schematic drawings of a single bract/scale complex in different taxodiaceous Cupressaceae (side view). (A–C) *Glyptostrobus pensilis*; (A) wild-type at pollination; (B) wild-type at seed maturity; (C) teratological bract/scale complex. In both (A and B), the ovule is axillary. In (C), the tooth forms an unusually strongly swollen bulge, the ovulate seed scale is recaulescent and fused to the lower sterile seed scale, causing dislocation of the ovule to the centre of the bulge so that the ovule is no longer axillary. (D) *Cunninghamia*, ovules in a single row attached to the tongue-like tips of the ovuliferous scale. (E) *Athrotaxis*, ovules in a single row attached to a strongly reduced ovuliferous scale. (F) *Sequoia*, ovules inserted in one or two rows; ovuliferous scale lacking free tips and entirely fused to the bract. Colours: yellow, cone axis; blue, primary fertile seed scale bearing the ovule; green, second sterile seed scale bearing the tooth; red, bract. Photo credit: V.M. Dörken.

The unusual central position of the ovules in the teratological cones, freely exposed on the teeth, is readily explained by a recaulescent fusion of the bract and the unusually strongly swollen seed scale and its intercalary growth (Fig. 6). Under this interpretation, following contact between the primordia of the fertile and sterile seed scales, the upper ovulate seed scale was shifted out of its originally axillary position by swelling of the lower sterile seed scale, resulting in dislocation of the ovule out of its axillary position. Thus, the teratological cone scales bear two axillary structures, which we interpret as descending accessory shoots (ovuliferous scales or seed scales). A similar feature occurs in non-taxodiaceous Cupressaceae, though in these cases all seed scales are fertile and ovule-bearing and reduced to the ovules and lack any visible vegetative parts of the shoot axis (Jagel & Dörken, 2015).

### Two descending shoots are inserted in the axil of each bract

Interpretation of the axillary teeth in the bract/scale complex of *Glyptostrobus* remains controversial. Their relatively late development, always significantly later than the ovules, strongly contradicts their interpretation as ovule-bearing structures. Thus, we propose their interpretation as two descending accessory shoots: a primary fertile shoot that bears the ovules and a secondary sterile one that bears the teeth, resulting in the apparently axillary position of ovules in wild-type seed cones. This scenario best explains the relatively late development of the teeth, always significantly later than the fertile row of ovules. It also explains the presence of a distinct ovuliferous tissue on the upper surface of the lower teeth in our teratological seed cone A (Figs. 5A and 5E; see discussion below).

Comparison with fossil Cupressaceae supports our interpretation that Cupressaceae possess a unique (potentially synapomorphic) proliferation of axillary structures in their cone scales. The extensive fossil history of *Glyptostrobus* displays a morphological stasis over a considerable period of geological time (Stockey et al., 2005; LePage, 2007). Examination of anatomically well-preserved cones displays few differences from living species (Matsumoto, Ohsawa & Nishida, 1997). On the other hand, our interpretation of the teeth as representing an axillary shoot in the sense of a sterile seed scale is supported by comparison with other fossil Cupressaceae; in contrast with most modern taxa, the earliest Cupressaceae also possessed a well-defined bract/scale complex (Escapa, Cúneo & Axsmith, 2008; Spencer et al., 2015). In *Scitistrobus duncaanensis*, the oldest known anatomically preserved Cupressaceae seed cone from the Middle Jurassic, a seed scale showing three free tips was inserted within the axil of its rhomboidal bract (Spencer et al., 2015). However, the seed scale was less conspicuous than the bract to which it was partly attached, diverging from the middle of the bract and becoming reflexed towards the cone axis. Three seeds were developed on each seed scale. In contrast with most extant Cupressaceae s.str., the ovules/seeds of *S. duncaanensis* were not axillary but rather attached to the upper surface of each free scale tip. Thus, the free tips of the seed scales as developed in *S. duncaanensis* are reminiscent of some extant Cupressaceae such as some *Cunninghamia* species (Serbet, Bomfleur & Rothwell, 2013; Spencer et al., 2015).

The bract/scale complexes in our teratological *G. pensilis* seed-cone A show also some similarities with extinct—but well-documented—fossil Voltziales, though with some important distinctions. Within all Voltziales, the ovules were not axillary, but instead were always located on the upper surface of the ovuliferous scale. Seed-scale shapes and number of ovules per scale were diverse within Voltziaceae (Florin, 1951; Sporne, 1965; Stewart & Rothwell, 1993; Taylor, Taylor & Krings, 2009). Rothwell et al. (2011) proposed a well-illustrated transition series for the evolution of the bract/scale complex in Cupressaceae derived from the extinct Palaeozoic Voltziales, commencing with slightly three-dimensional ovuliferous dwarf shoots that retained both vegetative scales and sporophylls. Progressive reduction of the bract/scale complexes in taxa such as *Hughmilleritis* and *Elatides* resulted in apparent attachment of the seeds to swollen ridges formed by the seed scale, but always distinctly below the free tips of the seed scale. In the final step of the reduction series, the free tips of the seed scale were entirely reduced and the seed scale was visible only as a small bulge in basal parts of the bract where the seeds are attached (Rothwell et al., 2011).

On the other hand, significant differences in vasculature are evident, not only between different species of Voltziales, but also between Voltziales and *Glyptostrobus*. In Voltziales, each bract and seed scale was supplied by a discrete vascular bundle that entered the stem vasculature of the cone axis as a separate strand. In *Pseudovoltzia*, the vasculature supplying the ovules clearly arose from the end of the seed-scale bundle (Florin, 1951; Sporne, 1965; Stewart & Rothwell, 1993). In contrast, in *Glyptostrobus* each ovule is supplied by a distinct vascular bundle strand that enters the stem vasculature separately and does not fuse with the bundle strands of the lower teeth and the bract before diverging from the cone axis (Fig. 1D). The *Glyptostrobus* ovules are not supplied by a vascular strand derived from the seed-scale strand, a critical difference from Voltziales.

### In teratological seed cones, a detached epidermal layer forms an outer ovule envelope

During recaulescent fusion of the fertile and sterile seed scales, the epidermal layer of the fertile upper seed scale envelops the ovule as a single layer of cells that becomes detached at distal regions close to the ovule (Figs. 4E, 4F, 5A, 5B and 5D–5F), probably due to the central dislocation of the ovule and its upward curvature as the tooth becomes swollen. This unusual detached epidermal layer was present in all cone scales of all teratological seed cones examined here. In most cases, this tissue fits closely over the ovule surface, but on some ovules it was extremely strongly developed and hence wrinkled (Fig. 4F). Thus, it represents a novel additional envelope around the ovule, though it always leaves the exposed micropyle clear so that pollination remains possible.

This unusual detached epidermal layer is potentially analogous with the outer integument, which is widely viewed as a novel structure of angiosperms (Stewart & Rothwell, 1993; Endress & Igersheim, 2000). However, although it could potentially play a similar role, it differs from the angiosperm outer integument in that it is uniseriate. Recent molecular-genetic studies of water lilies indicate that homologues of *INNER NO OUTER* (*INO*), a member of the YABBY gene family, are implicated in the evolution of the outer integument (Yamada, Ito & Kato, 2003). On the other hand, *INO* is expressed primarily in the abaxial region of the outer integument, and other genes are also involved (Kelley & Gasser, 2009). These differences indicate that although no direct homology can be inferred, this unexpected uniseriate teratological structure indicates one possible route to evolving an outer ovule envelope. Comparative studies of taxa with unusual ovule morphology can help to elucidate other potential pathways to integument evolution.

### Teratological apical proliferations are common in conifer seed cones

The apical vegetative proliferation described here in the most strongly proliferated seed cone (cone A: Figs. 4A and 4B) represents an elongation of the seed-cone apex of a type that has been described in several other conifers (reviewed by Bateman, Hilton & Rudall, 2011; Rudall et al., 2011). Indeed, proliferated seed cones are relatively common in conifers compared with rare instances of proliferated pollen cones. However, most recorded teratological apical vegetative proliferations are short and unbranched. The apical proliferation reported here is remarkable in bearing lateral branchlets of even the fifth order (Fig. 4A).

Such terminal proliferations occur frequently among taxodiaceous Cupressaceae, including *Cryptomeria*, *Cunninghamia* and *Metasequoia* (Neubauer, 1976; Dörken, 2011; Jagel & Dörken, 2015). In all typical (wild-type) seed cones, the apex is used up while forming the distal cone scales, whereas proliferated seed cones maintain a longer period of growth, usually as a vegetative shoot axis that bears typical green, sterile trophophylls, lacking any leaves that are transitional with the distal fertile cone scales (Fig. 4B).

Rudall et al. (2011) suggested that this relative abundance of proliferated seed cones provides additional support for the widespread hypothesis that the conifer seed cone is polyaxial and hence analogous (possibly homologous) with an inflorescence, and the seed-cone scale therefore homologous with an angiosperm flower (Florin, 1951; Schweitzer, 1963; Stützel & Röwekamp, 1997, 1999; Mundry, 2000). In angiosperms that produce racemose inflorescences, the apex maintains vegetative growth until it becomes exhausted; some other species are characterised by late vegetative proliferation of the inflorescence apex.

## Conclusion

Heritable teratological forms not only demonstrate that a particular mutation is possible but also facilitate studies across non-model species such as conifers. Conifers and other gymnosperms appear to be particularly prone to such spontaneous abnormalities (Bateman, Hilton & Rudall, 2011; Dörken, 2011; Dörken & Nimsch, 2017; Rudall et al., 2011). Such transformational mutations could potentially involve transference of function between homologous structures and could ultimately promote major (even saltational) evolutionary change (Bateman & DiMichele, 2002; Baum & Donoghue, 2002). The observed developmental plasticity may be at least partly due to the highly condensed, integrated nature of the modern coniferous seed cone, in which each cone scale is interpreted as a congenitally fused lateral axis putatively derived from fertile branches similar to those of extinct cordaites (Florin, 1951). Evidence in support of this hypothesis is largely drawn from comparison with fossils, but comparison of wildtype and mutant conifer cones provides further data.

For example, the *acrocona* mutant of *P. abies* (Pinaceae) bears atypical shoot-like structures in the axils of basal bracts, interpreted by Carlsbecker et al. (2013) as chimeras between ovuliferous scales and vegetative short shoots. Carlsbecker et al. (2013) also noted their similarity to the ovuliferous bract/scale complexes of Permian–Triassic fossil cones. They investigated expression patterns of *DEFICIENS AGAMOUS-LIKE* (*DAL*) genes belonging to the *AGAMOUS* (*AG*) clade, which includes genes that are active in determination of both floral meristem and organ identity in angiosperms. They found that in the wildtype of *P. abies*, *DAL14* is expressed only in the seed scale at early stages (later extending to the adaxial side of the bract), but it is not expressed in these chimeric organs in the mutant form, indicating that it could function to suppress meristematic activity in a more typical ovuliferous scale in this species. Teratological conifer seed cones in *Picea* can produce a proliferation of seed scales without each additional scale being subtended by a bract, suggesting that their absence may not be a major impediment to this interpretation.

However, Pinaceae are relatively distantly related to Cupressaceae among extant conifers (Gadek et al., 2000; Yang, Ran & Wang, 2012). Within the family Cupressaceae sensu lato, Groth et al. (2011) found little corresponding expression of *DAL* orthologs compared with Pinaceae seed-cone scales. Our observation of a mutant form of *G. pensilis* that features a remarkably distinct boundary layer between the ovulate tissue and the ovuliferous scale tissue is highly relevant in the context of understanding the homologies of conifer cones, and opens up the possibility of future gene-expression studies on similar mutants.

## Additional Information and Declarations

### Data Availability

The following information was supplied regarding data availability: The raw data are presented in the Figures.
